# Vaccination in the Light of the Royal British Commission

**Published:** 1898-04

**Authors:** M. R. Leverson

**Affiliations:** Fort Hamilton, L. I., N. Y.


### VACCINATION IN THE LIGHT OF THE ROYAL BRITISH COMMISSION.

M. R. Leverson, M. D., Fort Hamilton, L. I., N. Y.

(Continued from March No., p. 110.)

Qs. 4,721-2. Dr. Cory explained what he meant by the statement that a person vaccinated say last month could be re-vaccinated twelve months hence. The result from re-vaccination is a papule which varies from hardly anything at all to a papule of some size, and from that to a vesicle.

Q. 4,723. "Would you call that papule an effectual re-vaccination?" "Yes, I should certainly. It is as much as you can produce in that individual."

Q. 4,724 (By Sir James Paget). "It is a mere matter of words."

Q. 4,727 (By Dr. Bristowe). "You mean that these different papules and vesicles are the specific results of vaccination?" "That is what I mean." And (Q. 4,727a) "Though the specific result got in a case when there is only a papule is the only specific result you can possibly get, still it is specific."

Q. 4,728 (By Prof. Michael Foster). "That papule is the maximum effect which can possibly be produced in the subject at that time by any quantity of the virus?" "Yes."

Q. 4,729. "When the condition of the subject is changed then you may get a complete vesicle, owing to the diminution of the immunity?" "Yes."

Q. 4,730. "And that complete vesicle bears the same relation to the subject as your papule does at the earlier stage, where there is a certain larger quantity of immunity still left in the organism?" "Yes."

Q. 4,731. Prof. Foster suggests two different names for the two kinds of results, but Dr. Cory replies that it is in either case a successful re-vaccination, and that is all that you can certify.

Q. 4,732 (Mr. Meadows White). "You mean that being successful it is evidence of immunity, and that it is as much as you can get; and, therefore, it shows to you a satisfactory state of things, the patient being, according to your judgment, protected?" "Yes, fully protected by the re-vaccination."

Q. 4,733 (By Prof. Michael Foster). "Are you aware that in the minds of a great many people successful re-vaccination would mean that the immunity had been lost?" "Yes, no doubt; but the loss of the immunity is a gradual loss."

Q. 4,734. Dr. Cory says: "You might define successful re-vaccination as that amount of result that you would produce if a person would have caught small-pox on being exposed to it. But I do not thus define it."

Qs. 4,735-6. Where a complete vesicle running the course of a primary vesicle is produced on a re-vaccination, Dr. Cory believes that the individual, if he had been exposed to small-pox, would have taken it; but if a papule only had been produced he would not necessarily have been liable to small-pox if exposed to it.

Q. 4,737 (Prof. Michael Foster). "In the minds of most people successful vaccination means a vesicle, which in your mind means the liability to take small-pox?" "Yes, very likely."

Q. 4,738. Those people would not apply the phrase "successful vaccination" to the papule which Dr. Cory regards as indicating the protection to a certain extent against small-pox.

Q. 4,740 (By Mr. Savory). The difference between the vesicle of successful re-vaccination and that of primary is entirely a matter of degree.

Q. 4,742. "After successful re-vaccination could you tell the vesicle which follows from the vesicle of a primary vaccination?" "I believe in the majority of cases you could."

Q. 4,744. There is still some effect of the primary vaccination left. The vesicle of re-vaccination runs its course more rapidly than that of primary vaccination (Q. 4,746).

Q. 4,747. That fact implies that there was some effect of the primary vaccination still remaining.

Q. 4,748 (Prof. Michael Foster). "So that practically absolutely successful re-vaccinations as indicating that no trace has been left of the original vaccination are extremely rare?" "Extremely rare. There are few individuals that are liable to a second attack of small-pox, and there are about the same number, I should think, that are liable to fully successful re-vaccination."*

Q. 4,749 (Sir Edwin Galsworthy). "But is not a second attack of small-pox frequently as severe as the first?" "I believe it is."

Q. 4,750 (Dr. Collins). "Have you had any experience of vaccinating persons who have had small-pox?" "Yes, I have vaccinated several. A good many of the post-office boys who come up for re-vaccination are boys who have never been vaccinated before, but who have had small-pox." (Q. 4,751.) "The result is exactly like an ordinary re-vaccination."

Qs. 4,754-5. "Let us take two adults of like age, one of whom had small-pox at a certain date, while the other was vaccinated at the same date; if you were to re-vaccinate the latter and vaccinate the former the results would be the same. The person who was re-vaccinated would have a modified vesicle, and the one who had had small-pox would have a modified vesicle in the same form." The vesicles of those who have had small-pox present the same kind of differences of degree of susceptibility to the vaccine virus as in the case of re-vaccination. (Chairman.)

Q. 4,756. "All those who have had small-pox and are afterwards vaccinated do not present exactly the same characteristics?" "No; it will depend on the interval between their small-pox and their subsequent vaccination."

---

* The number of second, third, fourth, etc., attacks of small-pox is *greater* than on the doctrine of probabilities alone. It ought to be.

The assumption that small-pox is auto-protective against itself is an assumption unsupported by the facts. (M. R. L.)

Q. 4,757. You will find in some papules, in some vesicles.

Qs. 4,758-63. Has vaccinated about twenty persons after small-pox. None gave a vesicle.

Q. 4,764 (By Dr. Collins). There is not much difference among calves in susceptibility to vaccination. He thinks he has had but one unsusceptible calf out of 1,500.

Qs. 4,765-6. Re-vaccinating calves gives no result at all if there has been a successful primary vaccination.

Q. 4,768. Dr. Cory had forgotten what he had said in his thesis with regard to the raising of vaccine lymph from animals suffering from cattle plague, and could not state whether the results of such vaccinations were normal and successful or not.

Q. 4,769. In that thesis—speaking of Dr. Macpherson's experiments—he said that the vaccination of children from the crusts obtained from the cattle "had all the characters of true vaccine; the child suffered much from fever for four days. Two children were vaccinated from this vesicle with complete success, the symptomatic fever being very severe. From these two children five others were successfully vaccinated, and the stock thus established was afterwards regularly continued. Some of the children vaccinated with this lymph were tested by variolous inoculation, and exposed to variolous infection and found secure." "Have you formed any opinion as to those experiments of cattle plague inoculation?" "I know Dr. Murchison advocated the vaccination of animals to save them from the cattle plague, and there were a large number of experiments performed upon the animals at that time, but they did not save them at all from cattle plague."

Q. 4,770. "But I was referring to the inoculation of children with lymph obtained from that source?" "This is quite beyond my personal experience. This is only what I got from literature."

